# Reduced Cortisol Output during Public Speaking Stress in Ostracized Women

**DOI:** 10.3389/fpsyg.2017.00060

**Published:** 2017-02-08

**Authors:** Ulrike Weik, Jennifer Ruhweza, Renate Deinzer

**Affiliations:** Institute of Medical Psychology, Justus Liebig University GiessenGiessen, Germany

**Keywords:** acute stress, Cyberball ostracism, social exclusion, cortisol, hypothalamic-pituitary-adrenal-axis

## Abstract

Ostracism (being excluded or ignored) is experienced as unpleasant and distressing. In previous studies, an immediate pre-stress experience of ostracism induced by Cyberball, a virtual ball-tossing game, was found to inhibit cortisol reactivity to public speaking stress in female students. The present study examines whether the effect will persist when a 15-min time gap between the Cyberball experience and subsequent psychological stress is introduced. *N* = 84 women were randomly assigned to Cyberball ostracism vs. inclusion. 15 min after playing Cyberball, all women were subjected to public speaking stress. Salivary cortisol and mood were repeatedly assessed during the course of the experiment. These are the main findings of the study: Repeated measures ANCOVA revealed that public speaking stress resulted in a significant increase of cortisol in both groups (inclusion vs. ostracism). However, cortisol levels were significantly lower in the ostracism group. In earlier studies when Cyberball was played immediately before public speaking stress, the cortisol response to public speaking was completely suppressed in ostracized women. By introducing a waiting period between Cyberball and public speaking stress in the present study, the main effect of an ostracism induced reduction of cortisol remained, although both groups showed an increase of cortisol as a response to public speaking. These results again suggest that the experience of ostracism might inhibit hypothalamic-pituitary-adrenal (HPA) axis activity, thereby confirming previous results. The formerly observed total suppression of HPA axis responsiveness to public speaking, however, seems to be a rather short-term effect.

## Introduction

The need to belong, i.e., establishing and maintaining social relationships, is an existential need for human beings (Baumeister and Leary, 1995; Cacioppo et al., 2011). The experience of being ostracized (being socially excluded or ignored) or rejected is perceived as unpleasant and painful (Zadro et al., 2004; MacDonald and Leary, 2005). In experimental research several paradigms are used to induce social exclusion, ostracism or rejection (Nezlek et al., 1997; Twenge et al., 2001; Baumeister et al., 2002; for overview see also Williams, 2007; Blackhart et al., 2009). The Cyberball paradigm (virtual ball tossing game) was developed to specifically induce ostracism (Williams et al., 2000; Williams and Jarvis, 2006). According to the ostracism detection theory (Williams et al., 2000; Williams and Zadro, 2005; Williams, 2009), social beings possess a rapid detection system activated by any cue of being ignored or excluded. Detecting ostracism is considered to be adaptive, allowing (re-) establishment of social support and helping to be included again. In the last decade, Cyberball has gained much attention in psychological research as it allows studying the impact of ostracism or the experience of social exclusion on the individual’s psychological wellbeing in a highly standardized manner. A considerable number of studies provide evidence of short-term effects on mood, physiological arousal and behavior (Gerber and Wheeler, 2009; Williams, 2009). Furthermore, fundamental needs (e.g., belonging and control) are threatened by Cyberball ostracism. Research also indicates the involvement of brain areas associated with physical pain in the experience of being ostracized via Cyberball (Eisenberger et al., 2003).

The present study focuses on neuroendocrine responses to Cyberball ostracism. An important neuroendocrine stress response system is the hypothalamic-pituitary-adrenal (HPA) axis. During an acute stressful event, corticotrophin releasing factor is secreted from the paraventricular nucleus of the hypothalamus triggering the anterior pituitary to produce adrenocorticotrophin hormone (ACTH). ACTH stimulates the adrenal glands which in response releases the glucocorticoid hormone cortisol. Acute cortisol release is considered as adaptive as it supports the organism to maintain homeostasis by inducing release and distribution of energy stores (Chrousos and Gold, 1992; Sapolsky et al., 2000). A variety of experimental research has shown that a short term and time limited cortisol release can be induced by certain laboratory stress tasks. It was found that in particular uncontrollable and social-evaluative performance tasks are potent triggers of HPA-axis activation resulting in cortisol release (Dickerson and Kemeny, 2004).

Given the fact that being ostracized is considered to be a potent social stressor, it might be expected to be capable of triggering HPA-axis and consequently leading to a cortisol release. Surprisingly, results of studies investigating the direct effect of Cyberball ostracism on cortisol indicate that this is not the case (Zöller et al., 2010; Geniole et al., 2011; Zwolinski, 2012; Seidel et al., 2013). In order to understand these findings it might be helpful to consider the nature of the stressor. Stressors known to elicit cortisol responses typically contain a strong evaluative component. This mainly activates the need for power rather than other motives such as the need for achievement or affiliation (Wiemers et al., 2015). On the other hand, Cyberball ostracism first and foremost induces a state of not belonging to a group. Thereby, it is more prone to affect the need for affiliation than the need for power. Interestingly, although Cyberball ostracism does not induce cortisol release, it still appears to affect HPA-axis by altering cortisol responsiveness to subsequent stress, however, in an unexpected way.

When Cyberball was played immediately before a second stressor, i.e., public speaking, gender-specific alterations in the cortisol response to public speaking stress were found (Weik et al., 2010, 2013). Women who underwent the ostracism condition failed to mount a cortisol response to a post-ostracism laboratory speaking task, while women in the inclusion condition exhibited significant responses. These results indicate that even if there is no direct effect of Cyberball ostracism on cortisol secretion, it still may have neuroendocrine effects that become apparent in stressful situations after the experience of ostracism. In the context of the ostracism detection theory, these effects might mirror a reflexive physiological response to ostracism (Williams, 2009). In order to better understand these mechanisms, their temporal dynamics should be known first: Will the suppression of cortisol remain for a longer period of time or is it just a rather short term effect disappearing after a few minutes? The present study therefore examined whether the ostracism effect on cortisol will still be observable when public speaking stress is not applied immediately after the ostracism experience but after a short time lag (i.e., 15 min). Only women were analyzed as previous results on Cyberball induced suppression of cortisol response to public speaking stress are confined to women so far (Weik et al., 2010).

It was hypothesized that Cyberball ostracism would affect cortisol secretion in women subsequently subjected to a public speaking stress also under conditions of a 15-min waiting period between Cyberball and public speaking stress.

## Materials and Methods

### Study Participants and Ethics

Study participants were 84 healthy female students aged 20–30 years (inclusion criteria) who were recruited by postings on the campus and announcements in local magazines. Students interested in study participation were contacted by telephone. During this first contact they received information about the study and were asked about inclusion and exclusion criteria (according to our previous studies). Exclusion criteria were applied in accordance to previous work mainly in order to avoid any disturbances of the endocrine system or other factors which might affect results. They were: study of psychology or human medicine; acute or chronic infection, acute allergy, diseases of the adrenal or thyroid glands, any history of neurological or psychiatric diseases, as well as psychotherapeutic treatment, vaccination and donation of blood before study onset, pregnancy or breast feeding, nicotine consumption or an irregular menstrual cycle, any known acute or chronic stress (e.g., any kind of examination) from 2 weeks prior to until 1 week after the study. For study participation, participants received a small monetary compensation for their time investment.

From the total sample, one woman in the ostracism group had to be excluded from the analyses because at the end of the experiment she indicated that she had known Cyberball beforehand. One woman in the inclusion group was excluded due to withdrawal from further participation during the stress session. Five women in the inclusion group and eight women in the ostracism group were further excluded because saliva samples for baseline and/or during stress did not contain enough saliva for cortisol analysis. The final sample included 69 female students: inclusion (*n* = 36) and ostracism (*n* = 33).

The study was approved by the Ethics Committee of the Medical Faculty of the University of Duesseldorf, Germany (Study No. 2558) and was found to conform to the guidelines of the World Health Organization (Declaration of Helsinki). All participants gave their informed, written consent.

### Overall Procedure

Students interested in study participation were contacted by telephone to provide information about the study and check for inclusion and exclusion criteria. Eligible participants were invited for a first appointment, which for each participant took place at least 1 week prior to the experiments. At this first appointment, exclusion and inclusion criteria were assessed again and participants then received detailed oral and written information about the experiment before they provided their written consent. Additionally, they filled in the questionnaires assessing the control variables and they were photographed for the Cyberball game. Detailed instructions were also given with respect to behavioral rules for the day before and the day of the experiment (see section Control Procedures), which were also provided in written form.

**FIGURE 1:**
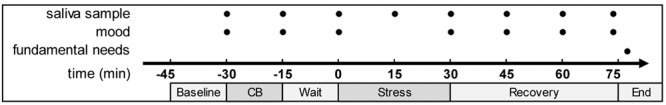
**Time points of measurement during the experimental periods** (Baseline: baseline period; CB: Cyberball; Wait: waiting period; Stress: public speaking stress; Recovery: stress recovery period; End: end of the experiment).

The experiments took place at the second appointment approximately 1 week after the first appointment. Time points of the experimental assessment of the dependent variables are shown in **Figure 1**. When arriving at the laboratory, participants were seated in a quiet room for acclimatization. After the baseline period, the participants were placed in front of a computer and they received verbal as well as written instructions with respect to Cyberball. Before starting the game, the experimenter led the participants to believe that she had to leave the room in order to check whether the other three players (computer generated) were ready to play. After the experimenter had returned, participants were told that all players were ready and the game could be started (see also Weik et al., 2013). After Cyberball, the participants remained seated for 15 min without any further instruction. Then, participants underwent public speaking stress in a separate room. Afterward they returned to the room in which baseline measures were assessed for the recovery period. They were instructed to remain seated and they had the opportunity to read comics or travel journals. At the end of the recovery period, threat to fundamental needs was assessed. A detailed debriefing of the participants by the respective experimenter completed the experiment.

### Experimental Conditions

#### Independent Variable: Cyberball Inclusion vs. Ostracism

In accordance with previous studies (Weik et al., 2010, 2013; Zöller et al., 2010), ostracism (social exclusion) was induced via the Cyberball paradigm (Williams and Jarvis, 2006). In the Cyberball game, participants are led to believe that they are playing a virtual ball-tossing game with three other players [in our case: one of the same and two of the opposite sex (Weik et al., 2010, 2013)]. In fact, activities of the three other players are computer-generated. Names and photographs of all players are displayed beneath the player’s icon on the computer screen. By mouse click on the photographs, the players can throw a ball to one of the other players. In total there were 60 ball throws. Two conditions were run in the present study: in the *ostracism condition* study participants received the ball three times, thereafter they did not receive it again; in the *inclusion condition* the participants received an average of every fourth ball.

Study participants were randomly assigned to one of the two experimental conditions and stratified with respect to oral contraceptive intake as well as menstrual cycle phase (follicular/luteal). The respective cycle phase of the participant on the day of the experiment was estimated on the basis of the information obtained by the anamnestic interview about cycle length and first day of the last menstruation.

As described previously, randomization was carried out by a person not involved in data assessment and not in contact with the participants (Weik et al., 2010, 2013; Zöller et al., 2010). The experimenter (JR) who was in direct contact with the participants was blinded until the end of the experiment, when participants were debriefed. Study participants were kept blinded with respect to hypotheses by a cover story (German translation of the cover story on the welcome page of the original Cyberball game: examining the effects of a mental visualization task performance on the endocrine system; see Williams and Jarvis, 2006).

#### Public Speaking Stress

The stress challenge took place in a separate room with one TV camera in front of a table with a chair on which the participants were seated. Two additional video cameras were placed at two upper, opposite corners of the room. All of the cameras were connected to an observation room equipped with a mixer desk and three observation monitors were connected to the cameras. This setting was visible to participants, who, in order to enter the room in which the stress challenge took place, walked across the observation room. The public speaking stressor has been described in detail previously (e.g., Deinzer et al., 2004; Weik et al., 2008). Briefly, it is a highly standardized stress paradigm consisting of three consecutive phases each lasting 10 min. In the first phase (anticipation) participants are only given brief information about the nature of the task. In the second phase (preparation) they are provided with details about the task (i.e., holding a speech in front of a camera with a given topic and specified quality criteria to be fulfilled). In the third phase (speech) they hold the speech and will be disturbed by the experimenter reminding them of the quality criteria to be fulfilled. In previous studies, this stress paradigm has been shown to be an effective psychological stressor consistently eliciting significant increases in salivary cortisol (Deinzer et al., 2004; Weik et al., 2008, 2010, 2013; Keitel et al., 2011; Schut et al., 2012) and ACTH concentrations (Weik et al., 2013). The sympathetic nervous system is affected in the same manner, as reflected by stressor induced increases of epinephrine and norepinephrine concentrations (Weik et al., 2013).

### Dependent Variables

#### Salivary Cortisol

In order to assess cortisol alterations throughout the experiment, eight saliva samples were collected by using Salivettes (Sarstedt, Nümbrecht, Germany) every 15 min from the beginning until the end of the experiment (**Figure 1**). The saliva samples were stored at -20°C until assayed. For cortisol analysis, samples were defreezed and centrifuged at 1700 × *g* for 5 min. Each sample was analyzed in duplicate. Salivary cortisol levels were determined by the use of commercial enzyme immunoassays (ELISA; IBL International^®^, Hamburg, Germany); intra- and inter-assay coefficients of variation were 3.1–7.3% and 6.4–8.8%, respectively.

#### Mood

In order to prove that Cyberball would alter mood and that a psychological response to public speaking would occur, participants’ mood ratings were assessed during the course of the respective experimental conditions (Cyberball inclusion vs. ostracism). The German short versions of the Multidimensional Mood Questionnaire (MDBF; Steyer et al., 1997) and the Differential Affect Scale (DAS; Merten and Krause, 1993) were used with internal consistencies (Cronbach’s α) of 0.73–0.89 and 0.54–0.80, respectively. The short version of the MDBF consists of 12 items assessing three factors: mood (good vs. bad mood), alertness (alertness vs. tiredness), and calmness (calmness vs. agitation). Three scales of the DAS assessing happiness, depression, and anger, each by three items, were used. Participants were instructed to rate the feelings they had during the preceding section of the experiment via visual analogue scales (VAS). The assessments took place in parallel to the collection of saliva samples except during the stress challenge (**Figure 1**). For all mood measures at each time point of measurement we found internal consistencies (Cronbach’s α) between 0.60 and 0.94.

### Manipulation Check

In order to prove whether the Cyberball manipulation was effective, feelings of exclusion were assessed by a VAS item immediately after Cyberball.

Furthermore, at the end of the experiment, threat to fundamental needs was assessed via a standard questionnaire in accordance with Zadro et al. (2004), consisting of 12 items assessing the effect of Cyberball on four needs: belonging, self-esteem, control and meaningful existence. Participants answered these questions by ratings on a 5-point scale, with 1 = not at all and 5 = very much. The internal consistencies (Cronbach’s α) for the fundamental need items found in the present sample were: belonging: 0.79; control: 0.80; meaningful existence: 0.57; self-esteem: 0.75.

### Control Variables and Control Procedures

#### Psychological Variables

To control for potential psychological confounders and for the respective comparability of the groups, the following variables were assessed by means of validated standardized questionnaires: Perceived social support (Questionnaire for the assessment of social support (Fragebogen zur sozialen Unterstützung; Fydrich et al., 2007)), Big Five personality factors (German version of the NEO-FFI; Borkenau and Ostendorf, 1993), Self-esteem (German version of the multidimensional concept scale (MSCS); Schütz and Sellin, 2006), Trait rumination (German version of the response styles questionnaire (RSQ-D); Kühner et al., 2007), and Locus of control (German version of the internal-external control scales (IPC); Krampen, 1981). Internal consistencies (Cronbach’s α) of these measures in the present sample were between 0.55 and 0.93.

#### Control Procedures

In order to control for circadian variations of cortisol secretion, all experiments took place between 2 and 4 pm. For the day before and the day of the experiment, the participants were asked to adhere to the following behavioral rules: to refrain from eating or drinking (except water) within 3 h prior to the beginning of the experiment and to refrain from physical activity (e.g., cycling to the laboratory) and medication intake on the day of the experiment. Furthermore, they were instructed not to drink any alcohol as well as to refrain from excessive physical activity on the day prior to the experimental days, to ensure at least 6 h of sleep at night and being awake at least 4 h prior to the experiment.

### Statistical Analysis

All statistics were computed using SPSS 22. The intended level of significance was α = 0.05.

Comparability of the ostracism vs. inclusion group with respect to baseline values of dependent and control variables was tested by χ^2^ tests and *t*-tests, as appropriate. In cases of significant group differences within the baseline or control variables, these variables were included as covariates in any further group comparison.

In order to prove whether the Cyberball manipulation was effective, groups were compared with respect to their feelings of exclusion immediately after Cyberball by univariate analysis of covariance (ANCOVA). Considering threat to fundamental needs, included and excluded women were compared by the means of multivariate analysis of covariance (MANCOVA).

To test for treatment effects on salivary cortisol concentrations and mood ratings, repeated measurement analyses with baseline cortisol and oral contraceptive intake included as covariates (repeated measurement ANCOVAs) were computed with group as between- and time points of measurement as within-factors. Baseline and control variables with significant group differences were included as further covariates in all group comparisons. According to one of the reviewers’ suggestions analyses were also run with all control variables as covariates. As this did not change results with respect to main effects of group and time these results are not reported here. Greenhouse-Geisser corrections were applied and original degrees of freedom together with Greenhouse-Geisser’s 𝜀 are reported. All ANCOVAs are presented with η^2^ as measure of effect sizes. Additionally, groups were compared at each time point of measurement by computing univariate ANCOVAs.

## Results

Inclusion (*n* = 36) and ostracism (*n* = 33) groups did not differ with respect to age (*t*_67_ = 0.966, *p* = 0.34; mean ± SD: 23.3 ± 1.7 and 22.9 ± 2, respectively) nor oral contraceptive intake and cycle phase (χ^2^ = 0.379, exact *p* = 0.86; follicular/luteal/oral contraceptives: 2/10/24 and 3/8/22). Significant differences were found with respect to self-related rumination (mean ± SD: 16.22 ± 4.1 and 14.12 ± 4.3, respectively; *t*_67_ = 2.079, *p* = 0.04) and distraction (mean ± SD: 18.72 ± 4.4 and 21.30 ± 4.9, respectively; *t*_67_ = -2.295, *p* = 0.03). No other group differences with respect to control variables were found (all *p* > 0.20).

**Table 1** shows descriptive data of baseline values and results of respective group comparisons. Groups differed significantly in their anger ratings at baseline (*t*_67_ = -2.847, *p* = 0.01). **Table 2** depicts Pearson and Spearman rank correlation coefficients of baseline variables.

**Table 1:** Group comparisons with respect to baseline variables.

	*M* (*SD*)		*t*	*p*
	Inclusion (*n* = 36)	Ostracism (*n* = 33)		
Cortisol (nmol/l)	7.10 (2.6)	6.60 (2.9)	0.801	0.43
Good mood	81.00 (11.5)	79.14 (13.9)	0.608	0.55
Calmness	69.52 (15.8)	73.69 (17.7)	-1.037	0.30
Alertness	64.94 (18.5)	64.5 (21.1)	0.092	0.93
Happiness	57.82 (17.0)	53.0 (17.7)	1.159	0.25
Depression	8.08 (8.7)	8.30 (9.2)	-0.102	0.92
Anger	3.71 (4.0)	7.80 (7.5)	-2.847	0.01

**Table 2:** Correlations of baseline variables (*N* = 69).

	Cortisol	Good mood	Calmness	Alertness	Happiness	Depression
Good mood	-0.18 (-0.19)					
Calmness	-0.12 (-0.13)	**0.48 (0.54)**				
Alertness	0.06 (0.07)	**0.47 (0.53)**	**0.26 (0.32)**			
Happiness	-0.18 (-0.06)	**0.35 (0.45)**	0.17 (0.17)	**0.46 (0.51)**		
Depression	**0.25 (0.24)**	**-0.53 (-0.41)**	**-0.33 (-0.32)**	-0.24 (-0.19)	-0.16 (-0.11)	
Anger	0.20 (0.19)	**-0.45 (-0.43)**	**-0.40 (-0.30)**	-0.19 (-0.17)	-0.23 (-0.24)	**0.53 (0.65)**

*Pearson correlational coefficients (*r*); [Spearman correlational coefficients (rho) in parenthesis]. Significant (*p* < 0.05) correlations are marked in bold.*

### Manipulation Check

Immediately after Cyberball, included and ostracized women differed significantly with more feelings of exclusion within the ostracism group (mean ± SD for inclusion and ostracism: 7.54 ± 12.03 and 52.14 ± 33.31, respectively; *F*_1/64_ = 52.768, *p* < 0.001, η^2^ = 0.45). With respect to fundamental needs, ostracized women reported higher need threat (mean ± SD for inclusion vs. ostracism, respectively: belonging: 3.77 ± 0.69 vs. 1.84 ± 0.60; self-esteem: 4.31 ± 0.54 vs. 3.10 ± 0.90; control: 3.41 ± 0.70 vs. 1.67 ± 0.58; meaningful existence: 4.10 ± 0.58 vs. 2.74 ± 0.74). MANCOVA revealed a highly significant group difference (*F*_4/61_ = 34.03; *p* < 0.001, η^2^ = 0.69).

### Mood Alterations

**FIGURE 2:**
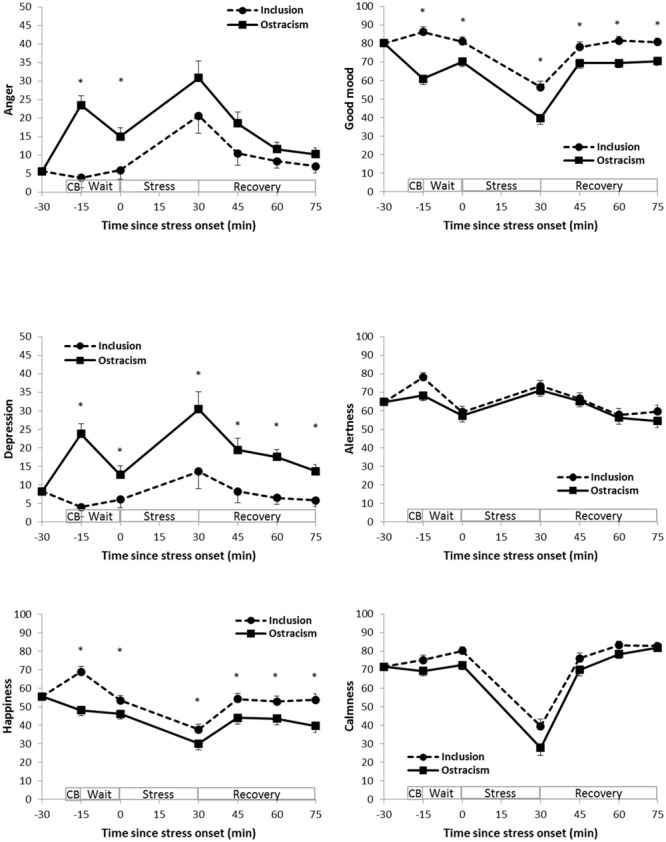
**Adjusted means and standard errors of the adjusted means of mood ratings (Differential Affect Scale: Anger, Depression and Happiness; Multidimensional Mood Questionnaire: Good mood – bad mood, Alertness – tiredness, Calmness – agitation).** ^∗^ significant (*p* < 0.05) group differences at single time points of measurement. CB: Cyberball; Wait: waiting period; Stress: public speaking stress. The adjustment procedure controls for baseline differences, oral contraceptive intake as well as for significant differences in control variables. It results in equal adjusted means at baseline.

Mood scores during the course of the experiment are illustrated in **Figure 2**. More negative ratings were observed in the ostracized compared to included group for the respective ratings for *anger* (time: *F*_5/315_ = 1.525, *p* = 0.21, η^2^ = 0.02, 𝜀 = 0.58; time^∗^group: *F*_5/315_ = 2.778; *p* = 0.04, η^2^ = 0.04, 𝜀 = 0.58; group: *F*_1/63_ = 9.850; *p* = 0.003, η^2^ = 0.14), *depression* (time: *F*_5/310_ = 1.371, *p* = 0.25, η^2^ = 0.02, 𝜀 = 0.60; time^∗^group: *F*_5/310_ = 2.972; *p* = 0.03, η^2^ = 0.05, 𝜀 = 0.60; group: *F*_1/62_ = 19.116; *p* < 0.001, η^2^ = 0.24), *happiness* (time: *F*_5/310_ = 2.318, *p* = 0.07, η^2^ = 0.04, 𝜀 = 0.72; time^∗^group: *F*_5/310_ = 1.978; *p* = 0.11, η^2^ = 0.03, 𝜀 = 0.72; group: *F*_1/62_ = 12.560; *p* = 0.001, η^2^ = 0.17), *good mood* (time: *F*_5/310_ = 3.174, *p* = 0.02, η^2^ = 0.05, 𝜀 = 0.78; time^∗^group: *F*_5/310_ = 3.954; *p* = 0.004, η^2^ = 0.06, 𝜀 = 0.78; group: *F*_1/62_ = 21.813; *p* < 0.001, η^2^ = 0.26) and *calmness* (time: *F*_5/310_ = 4.476, *p* = 0.003, η^2^ = 0.07, 𝜀 = 0.66; time^∗^group: *F*_5/310_ = 0.961; *p* = 0.42, η^2^ = 0.02, 𝜀 = 0.66; group: *F*_1/62_ = 5.056; *p* = 0.03, η^2^ = 0.08). No significant effects were found for *alertness* (time: *F*_5/310_ = 0.312, *p* = 0.86, η^2^ = 0.01, 𝜀 = 0.75; time^∗^group: *F*_5/310_ = 0.816; *p* = 0.51, η^2^ = 0.01, 𝜀 = 0.75; group: *F*_1/62_ = 1.202; *p* = 0.28, η^2^ = 0.02). Significant group differences for the respective single time points of measurement are indicated in **Figure 2**.

### Cortisol Alterations

**FIGURE 3:**
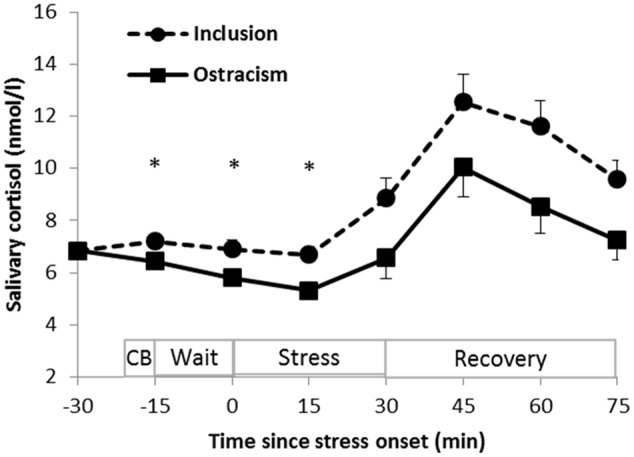
**Adjusted means and standard errors of the adjusted means of salivary cortisol concentrations during the experiment [time: *p* < 0.001; group: *p* = 0.027; ^∗^ significant (*p* < 0.05) group differences at the single time points of measurement].** CB: Cyberball; Wait: waiting period; Stress: public speaking stress. The adjustment procedure controls for baseline differences, oral contraceptive intake as well as for significant differences in control variables. It results in equal adjusted means at baseline.

Salivary cortisol concentrations at the respective time points of measurement during the course of the experiment are shown in **Figure 3**. Repeated measures ANCOVA revealed a significant main effect of time (*F*_6/372_ = 11.752, *p* < 0.001, η^2^ = 0.16, 𝜀 = 0.28) indicating that both groups showed a cortisol response to public speaking. However, overall cortisol levels remained lower in the ostracism group from the beginning of the waiting period until 45 min after public speaking (main effect group: *F*_1/62_ = 5.113, *p* = 0.027, η^2^ = 0.08). The time^∗^group interaction effect was not statistically significant (*F*_6/372_ = 1.206, *p* = 0.298, η^2^ = 0.02, 𝜀 = 0.28). *Post hoc* analyses for the respective single time points of measurements revealed that the most significant differences were found at the beginning of the waiting period (-15 min; *F*_1/64_ = 6.948; *p* = 0.01, η^2^ = 0.10), immediately after the waiting period (0 min; *F*_1/64_ = 7.335; *p* = 0.009, η^2^ = 0.10) and in the middle of the stress session (15 min; *F*_1/64_ = 10.433; *p* = 0.002, η^2^ = 0.14). Afterward increasing standard deviations led to less profound statistical effects though mean differences rather increased than decreased: at the end of the stress task (30 min; *F*_1/64_ = 2.631; *p* = 0.11, η^2^ = 0.04) and within the recovery period (45 min: *F*_1/64_ = 0.383; *p* = 0.54, η^2^ = 0.01; 60 min: *F*_1/64_ = 1.177; *p* = 0.29, η^2^ = 0.02; and 75 min: *F*_1/64_ = 2.038; *p* = 0.16, η^2^ = 0.03).

## Discussion

Consistent with previous work, the Cyberball paradigm clearly induced feelings of ostracism. Ostracism had an immediate effect on mood, particularly with respect to depression and anger ratings. In addition, ostracized women perceived themselves as being excluded and experienced threat to fundamental needs (see also Weik et al., 2010, 2013; Zöller et al., 2010). In line with previous results (Weik et al., 2010, 2013) the endocrine effects also appear to be contradictive at a first glance: Considering the absolute amount of cortisol secreted, reduced levels in ostracized as compared to included women are found. The reduction in cortisol in the current study is already observable immediately after Cyberball and continues during the public speaking stress. A similar tendency of subtle, though not significant, declines from baseline to after Cyberball has been observed before (Weik et al., 2010, 2013; Zöller et al., 2010).

However, in previous experiments, when the public speaking stress paradigm began immediately after Cyberball, ostracized women experienced not only a reduction in cortisol secretion, but indeed showed no cortisol response to public speaking stress at all. In the present study, though again a reduction in cortisol was observed in ostracized compared to included women, cortisol reactivity (in terms of increases in cortisol values) to public speaking stress was not affected any more (Weik et al., 2010, 2013). It thus appears that the introduction of the 15-min waiting period allowed the HPA-axis reactivity to recover. These results imply that the previously observed ostracism-induced suppression of HPA-axis reactivity to public speaking stress mirrors an immediate and rather short-lasting effect confined to Williams’ first, reflexive stage of ostracism effects (Williams, 2009). However, the experience of ostracism still seems to reduce total cortisol secretion.

Our results converge with findings of two other studies showing significant declines in cortisol levels after ostracism (Bass et al., 2014; Jobst et al., 2015) but findings in this research field are inconsistent. Several studies report no significant differences between ostracized and included participants (Zöller et al., 2010; Geniole et al., 2011; Zwolinski, 2012; Seidel et al., 2013). Interestingly, most studies tested the hypothesis that Cyberball ostracism would lead to cortisol increases. Some authors even appear to trust so much in this hypothesis that they refer to our results of cortisol non-responsiveness after ostracism as examples of enhanced responsiveness (Iﬄand et al., 2014; Beekman et al., 2016). Considering that Cyberball ostracism can be regarded as a potent social stressor, this misconception is partly comprehensible. Social evaluative threat is considered a robust and strong trigger of cortisol increases (Dickerson and Kemeny, 2004) and one might believe that Cyberball ostracism meets this criterion. However, Cyberball addresses foremost the need for affiliation and not the need for power as typical cortisol eliciting stressors do (Wiemers et al., 2015). Indeed, it has been shown that the strength of the affiliation motive is negatively associated with cortisol responses to acute stress (Wegner et al., 2014). Furthermore, with regard to stressors activating the need for affiliation, other stress regulatory systems have been suggested to be of high priority, particularly for females (Taylor et al., 2000). In this theory, the role of neuropeptides such as oxytocin and endogenous opioids promoting affiliative behavior is emphasized. Oxytocin is known for its potent anti-stress effects and for diminishing cortisol release under stress (Meyer-Lindenberg et al., 2011). The involvement of central pathways into ostracism experience that are known to decrease HPA axis activation have already been discussed by other authors (Bass et al., 2014; Jobst et al., 2015). Thus, the central release of oxytocin and the activation of further inhibiting pathways as a response to ostracism might account for the present results. Further studies are now needed to elucidate the specific role of these pathways in ostracism-induced reductions of cortisol release. The present study is not only of interest for bringing about new hypotheses regarding psychological and physiological mechanisms mediating between ostracism and stress responsiveness. It should be also noted that stress protocols eliciting cortisol responses are often characterized by the rather reserved and aloof behavior of the experimenter(s) when interacting with the study participants as described e.g., in Deinzer et al. (2004) and Frisch et al. (2015). This could represent a social cue inducing an experience of ostracism and the responses to it. From a methodological viewpoint, this might be important especially when it comes to the study of the stress responses of women, which are known to respond with high sensitivity to ostracism signals. One might hypothesize that the often observed reduced responsiveness of women compared to men in these classic stress paradigms might be partly due to this experience.

While the present study has its strengths in a well-controlled design with a considerable number of participants it has also some limitations. Though groups were comparable with respect to a variety of control and baseline variables indicating successful randomization, significant differences for the control variables self-related rumination and distraction as well as for baseline anger ratings were found which had to be controlled for by statistical means. While the study gives information about the effects of a 15-min time lag between ostracism and public speaking stress, information about longer and shorter time gaps is missing. Moreover it should be kept in mind that the present study sample comprises only women and thereby addresses women’s responses to Cyberball, only. It would be important to see if similar effects were observable for men. However, a previous study assessing the immediate effect of Cyberball on cortisol responses to public speaking stress did not find any Cyberball effect in men with respect to cortisol though women in the ostracism group showed a suppressed cortisol response (Weik et al., 2010).

In summary, the finding of the current study provides further support for the hypothesis that Cyberball ostracism exhibits a temporal inhibiting effect on subsequent cortisol release. Whereas former studies showed that an immediate sequence of Cyberball and acute stress leads to an inhibition of HPA axis reactivity to acute stress, the present finding indicates that this interference seems to be a short-term effect. With a time gap of 15 min between Cyberball and stress, HPA axis reactivity to stress is no longer affected. Still, ostracism results in lower overall cortisol output.

## Author Contributions

UW: conceptual design, supervision of and participation in data collection, cortisol analysis, analytic strategy, statistical analysis, and writing the paper. JR: contributions to the conceptual design, statistical analysis, writing the paper, data collection, and cortisol analysis. RD: conceptual design and overall conceptualization, supervision of data collection and analysis, analytic strategy, critical revision of manuscript drafts. All authors read and approved the manuscript.

## Conflict of Interest Statement

The authors declare that the research was conducted in the absence of any commercial or financial relationships that could be construed as a potential conflict of interest.

## References

[B1] Bass E. C. Stednitz S. J. Simonson K. Shen T. Gahtan E. (2014). Physiological stress reactivity and empathy following social exclusion: a test of the defensive emotional analgesia hypothesis. *Soc. Neurosci.* 9 504–513. 10.1080/17470919.2014.929533 24933604

[B2] Baumeister R. F. Leary M. R. (1995). The need to belong: desire for interpersonal attachments as a fundamental human motivation. *Psychol. Bull.* 117 497–529. 10.1037/0033-2909.117.3.497 7777651

[B3] Baumeister R. F. Twenge J. M. Nuss C. K. (2002). Effects of social exclusion on cognitive processes: anticipated aloneness reduces intelligent thought. *J. Pers. Soc. Psychol.* 83 817–827. 10.1037/0022-3514.83.4.817 12374437

[B4] Beekman J. B. Stock M. L. Marcus T. (2016). Need to belong, not rejection sensitivity, moderates cortisol response, self-reported stress, and negative affect following social exclusion. *J. Soc. Psychol.* 156 131–138. 10.1080/00224545.2015.1071767 26176743

[B5] Blackhart G. C. Nelson B. C. Knowles M. L. Baumeister R. F. (2009). Rejection elicits emotional reactions but neither causes immediate distress nor lowers self-esteem: a meta-analytic review of 192 studies on social exclusion. *Pers. Soc. Psychol. Rev.* 13 269–309. 10.1111/j.1745-6924.2009.01159.x 19770347

[B6] Borkenau P. Ostendorf F. (1993). *NEO-Five-Factors Inventory (NEO-FFI)*. Goettingen: Hogrefe.

[B7] Cacioppo J. T. Hawkley L. C. Norman G. J. Berntson G. G. (2011). Social isolation. *Ann. N. Y. Acad. Sci.* 1231 17–22. 10.1111/j.1749-6632.2011.06028.x 21651565 PMC3166409

[B8] Chrousos G. P. Gold P. W. (1992). The concepts of stress and stress system disorders. Overview of physical and behavioral homeostasis. *JAMA* 267 1244–1252. 10.1001/jama.1992.03480090092034 1538563

[B9] Deinzer R. Granrath N. Stuhl H. Twork L. Idel H. Waschul B. (2004). Acute stress effects on il-1β responses to pathogens in a human in vivo model. *Brain Behav. Immun.* 18 458–467. 10.1016/j.bbi.2003.11.008 15265539

[B10] Dickerson S. S. Kemeny M. E. (2004). Acute stressors and cortisol responses: a theoretical integration and synthesis of laboratory research. *Psychol. Bull.* 130 355–391. 10.1037/0033-2909.130.3.355 15122924

[B11] Eisenberger N. I. Lieberman M. D. Williams K. D. (2003). Does rejection hurt? An fMRI study of social exclusion. *Science* 302 290–292. 10.1126/science.1170008 14551436

[B12] Frisch J. U. Häusser J. A. Mojzisch A. (2015). The trier social stress test as a paradigm to study how people respond to threat in social interactions. *Front. Psychol.* 6:14 10.3389/fpsyg.2015.00014 PMC4313597 25698987

[B13] Fydrich T. Sommer G. Brähler E. (2007). *Fragebogen zur Sozialen Unterstützung (F-SozU)*. Goettingen: Hogrefe.

[B14] Geniole S. N. Carréa J. M. McCormick C. M. (2011). State, not trait, neuroendocrine function predicts costly reactive aggression in men after social exclusion and inclusion. *Biol. Psychol.* 87 137–145. 10.1016/j.biopsycho.2011.02.020 21382439

[B15] Gerber J. Wheeler L. (2009). On being rejected: a meta-analysis of experimental research on rejection. *Perspect. Psychol. Sci.* 4 468–488. 10.1111/j.1745-6924.2009 26162220

[B16] Iﬄand B. Sansen L. M. Catani C. Neuner F. (2014). Rapid heartbeat, but dry palms: reactions of heart rate and skin conductance levels to social rejection. *Front. Psychol.* 5:956 10.3389/fpsyg.2014.00956 PMC4148623 25221535

[B17] Jobst A. Sabass L. Palagyi A. Bauriedl-Schmidt C. Mauer M. C. Sarubin N. (2015). Effects of social exclusion on emotions and oxytocin and cortisol levels in patients with chronic depression. *J. Psychiatr. Res.* 60 170–177. 10.1016/j.jpsychires.2014.11.001 25466833

[B18] Keitel A. Ringleb M. Schwartges I. Weik U. Picker O. Stockhorst U. (2011). Endocrine and psychological stress responses in a simulated emergency situation. *Psychoneuroendocrinology* 36 98–108. 10.1016/j.psyneuen.2010.06.011 20650570

[B19] Krampen G. (1981). *IPC-Fragebogen zur Kontrollueberzeugung.* Goettingen: Hogrefe.

[B20] Kühner C. Huffziger S. Nolen-Hoeksema S. (2007). *Response Styles Questionnaire: RSQ-D*. Goettingen: Hogrefe.

[B21] MacDonald G. Leary M. R. (2005). Why does social exclusion hurt? The relationship between social and physical pain. *Psychol. Bull.* 131 202–223. 10.1037/0033-2909.131.2.202 15740417

[B22] Merten J. Krause R. (1993). *DAS (Differentielle Affekt Skala)*. Saarbrücken: Universität des Saarlandes.

[B23] Meyer-Lindenberg A. Domes G. Kirsch P. Heinrichs M. (2011). Oxytocin and vasopressin in the human brain: social neuropeptides for translational medicine. *Nat. Rev. Neurosci.* 12 524–538. 10.1038/nrn3044 21852800

[B24] Nezlek J. B. Kowalski R. M. Leary M. R. Blevins T. Holgate S. (1997). Personality moderators of reactions to interpersonal rejection: depression and trait self-esteem. *Pers. Soc. Psychol. Bull.* 23 1235–1244. 10.1177/01461672972312001

[B25] Sapolsky R. M. Romero L. M. Munck A. U. (2000). How do glucocorticoids influence stress responses? Integrating permissive, suppressive, stimulatory, and preparative actions. *Endocr. Rev.* 21 55–89. 10.1210/edrv.21.1.0389 10696570

[B26] Schut C. Weik U. Tews N. Gieler U. Deinzer R. Kupfer J. (2012). Psychophysiological effects of stress management in patients with atopic dermatitis: a randomized controlled trial. *Acta Derm. Venereol.* 93 57–61. 10.2340/00015555-1415 22983681

[B27] Schütz A. Sellin I. (2006). *Multidimensionale Selbstwertskala.* Goettingen: Hogrefe.

[B28] Seidel E. M. Silani G. Metzler H. Thaler H. Lamm C. Gur R. C. (2013). The impact of social exclusion vs. inclusion on subjective and hormonal reactions in females and males. *Psychoneuroendocrinology* 38 2925–2932. 10.1016/j.psyneuen.2013.07.021 23972943 PMC3863951

[B29] Steyer R. Schwenkmezger P. Notz P. Eid M. (1997). *Der Mehrdimensionale Befindlichkeitsfragebogen (MDBF)*. Goettingen: Hogrefe.

[B30] Taylor S. E. Klein L. C. Lewis B. P. Gruenewald T. L. Gurung R. A. Updegraff J. A. (2000). Biobehavioral responses to stress in females: tend-and-befriend, not fight-or-flight. *Psychol. Rev.* 107 411–429. 10.1037/0033-295X.107.3.411 10941275

[B31] Twenge J. M. Baumeister R. F. Tice D. M. Stucke T. S. (2001). If you can’t join them, beat them: effects of social exclusion on aggressive behavior. *J. Pers. Soc. Psychol.* 81 1058–1069. 10.1037/0022-3514.81.6.1058 11761307

[B32] Wegner M. Schüler J. Budde H. (2014). The implicit affiliation motive moderates cortisol responses to acute psychosocial stress in high school students. *Psychoneuroendocrinology* 48 162–168. 10.1016/j.psyneuen.2014.06.013 25016451

[B33] Weik U. Herforth A. Kolb-Bachofen V. Deinzer R. (2008). Acute stress induces proinflammatory signaling at chronic inflammation sites. *Psychosom. Med.* 70 906–912. 10.1097/PSY.0b013e3181835bf3 18799429

[B34] Weik U. Kuepper Y. Hennig J. Deinzer R. (2013). Effects of pre-experience of social exclusion on hypothalamus-pituitary-adrenal axis and catecholaminergic responsiveness to public speaking stress. *PLoS ONE* 8:e60433 10.1371/journal.pone.0060433 PMC3616100 23573255

[B35] Weik U. Maroof P. Zöller C. Deinzer R. (2010). Pre-experience of social exclusion suppresses cortisol response to psychosocial stress in women but not in men. *Horm. Behav.* 58 891–897. 10.1016/j.yhbeh.2010.08.018 20816966

[B36] Wiemers U. S. Schultheiss O. C. Wolf O. T. (2015). Public speaking in front of an unreceptive audience increases implicit power motivation and its endocrine arousal signature. *Horm. Behav.* 71 69–74. 10.1016/j.yhbeh.2015.04.007 25913901

[B37] Williams K. D. (2007). Ostracism. *Annu. Rev. Psychol.* 58 425–452. 10.1146/annurev.psych.58.110405.085641 16968209

[B38] Williams K. D. (2009). Ostracism: a temporal need-threat model. *Adv. Exp. Soc. Psychol.* 41 275–314. 10.1016/S0065-2601(08)00406-1

[B39] Williams K. D. Cheung C. K. Choi W. (2000). Cyberostracism: effects of being ignored over the Internet. *J. Pers. Soc. Psychol.* 79 748–762. 10.1037/0022-3514.79.5.748 11079239

[B40] Williams K. D. Jarvis B. (2006). Cyberball: a program for use in research on interpersonal ostracism and acceptance. *Behav. Res. Methods* 38 174–180. 10.3758/bf03192765 16817529

[B41] Williams K. D. Zadro L. (2005). “Ostracism: the indiscriminate early detection system,” in *The Social Outcast: Ostracism, Social Exclusion, Rejection, and Bullying* eds Williams K. D. Forgas J. P. von Hippel W. (New York, NY: Psychology Press) 19–34.

[B42] Zadro L. Williams K. D. Richardson R. (2004). How low can you go? Ostracism by a computer is sufficient to lower self-reported levels of belonging, control, self-esteem, and meaningful existence. *J. Exp. Soc. Psychol.* 40 560–567. 10.1016/j.jesp.2003.11.006

[B43] Zöller C. Maroof P. Weik U. Deinzer R. (2010). No effect of social exclusion on salivary cortisol secretion in women in a randomized controlled study. *Psychoneuroendocrinology* 35 1294–1298. 10.1016/j.psyneuen.2010.02.019 20334980

[B44] Zwolinski J. (2012). Psychological and neuroendocrine reactivity to ostracism. *Aggress. Behav.* 38 108–125. 10.1002/ab.2141 22331583

